# Differential chromatin profiles partially determine transcription factor binding

**DOI:** 10.1371/journal.pone.0179411

**Published:** 2017-07-13

**Authors:** Rujian Chen, David K. Gifford

**Affiliations:** Computer Science and Artificial Intelligence Laboratory, Massachusetts Institute of Technology, Cambridge, Massachusetts, United States of America; Università degli Studi di Milano, ITALY

## Abstract

We characterize how genomic variants that alter chromatin accessibility influence regulatory factor binding with a new method called DeltaBind that predicts condition specific factor binding more accurately than other methods based on DNase-seq data. Using DeltaBind and DNase-seq experiments we predicted the differential binding of 18 factors in K562 and GM12878 cells with an average precision of 28% at 10% recall, with the prediction of individual factors ranging from 5% to 65% precision. We further found that genome variants that alter chromatin accessibility are not necessarily predictive of altering proximal factor binding. Taken together these findings suggest that DNase-seq or ATAC-seq Quantitative Trait Loci (dsQTLs), while important, must be considered in a broader context to establish causality for phenotypic changes.

## Introduction

Differential transcription factor occupancy offers great insights into regulatory and developmental differences between cell states and cell types [1,2]. Chromatin immunoprecipitation and sequencing (ChIP-seq) [3] is a widely used approach to study the occupancy of factors of interest. More recently, chromatin accessibility assays such as DNase-seq and ATAC-seq has attracted interest as an alternative indicator of factor occupancy that does not require a separate experiment for each factor. It has been shown that in a single cell type, DNase-seq data can be used to predict ChIP-seq binding events for certain factors [4,5]. However, the ability of DNase-seq data to predict differential factor binding in different conditions has not been comprehensively studied.

Several methods have been developed to infer transcription factor binding from chromatin accessibility data. Centipede [4] uses a Bayesian hierarchical model for DNase data to infer bound and unbound sites. PIQ [5] uses a discriminative model to detect bound sites from unbound sites. The key feature and strength of both methods is the integration of sequence information (PWMs) and factor specific chromatin accessibility profiles. Centipede and PIQ achieved mean AUROCs (area under receiver-operating curve) of 0.87 and 0.93 respectively in predicting factor binding from DNase-seq data using binding events from 303 matched ChIP-seq data as held out labels for scoring [5].

Here we investigate if DNase-seq or ATAC-seq data from two conditions can be used to predict differential transcription factor binding in two conditions. We show that a naïve adaptation of existing methods is inadequate for the differential binding task, and propose a new method, DeltaBind, which extends PIQ to differential binding in a principled manner.

We examined how well DNase-seq data can predict where a transcription factor is bound in K562 cells and unbound in GM12878 cells for 18 distinct factors. We used cell-state matched ChIP-seq experiments to determine the differential ground truth binding for each factor. We evaluated several approaches for the inference of differential binding from DNase-seq data, and we found that methods based on DNase spatial read profiles are more effective than those based on aggregate read counts. In addition, jointly modeling the binding probabilities from both cell types further improves prediction accuracy. Based on these observations, we developed a general unsupervised method called DeltaBind to infer differential binding that outperforms other approaches for this task.

We found that the typical number of differential ChIP-seq events between K562 and GM12878 cells is very small. Averaged across all eighteen factors we studied, only 400 out of every 100,000 candidate binding sites (motif occurrences) are differentially bound (0.4%, range 0.1%– 1.3%). Therefore, the positive and negative sets in this inference task are extremely imbalanced, and a random predictor would only have an average precision or PPV (positive predictive value) of 0.4% and AUPR (area under precision-recall curve) of 0.004.

Using DeltaBind we are able to predict differential binding with an average precision of 28% (at 10% recall) and an AUPR of 0.127. Among the factors we studied, prediction accuracies for individual factors vary widely, ranging from 5% up to 65% precision, showing that some factors can be reasonably well predicted while some cannot. We find that a class of transcription factors called settler and migrant factors [5] generally have higher prediction accuracy, while for their counterpart, the pioneer factors, DNase read profiles have less predictive power for differential factor binding.

The above findings on the predictability of differential binding from chromatin accessibility profiles can also be observed in other settings. We present statistics from a study of differential CTCF occupancy and their associated DNase-seq signals at single nucleotide polymorphism (SNP) sites, where we observe a similar level of differential binding predictability to what we found in our experiments.

Taken together, our results suggest that chromatin accessibility information, while important, can only partially establish differential binding for individual factors across cell states, with an accuracy that is factor specific. In general, additional genomic data will need to be considered improve the prediction of differential factor binding.

## Results

### DNase-seq read counts are a poor predictor of CTCF occupancy at CTCF motifs that contain SNPs

Using data from a study of 114 cell and tissue types from 166 individuals [6] we examined the ability of DNase-seq data to predict CTCF occupancy at CTCF binding sites where the two alleles differ by a single nucleotide polymorphism (SNP). Of the 11355 CTCF sites in the study that contained an allelic SNP across all individuals, 810 (7%) of the CTCF sites exhibited differential CTCF binding, and 3079 (27%) had differential read count DNase-seq signal. Of the 8276 sites that did not exhibit DNase-seq read imbalance, 8032 (97%) had no ChIP-seq differential binding. However, of the 3079 sites that had differential DNase-seq signal, only 566 (18%) exhibited differential CTCF binding. Thus, DNase-seq imbalance does not necessarily establish differential binding (18% precision).

### Accuracy of differential occupancy detection varies among factors

We evaluated the ability of DeltaBind and DNase-seq data to predict factors that are bound in K562 and unbound in GM12878 cells for 18 different transcription factors (see Methods). Following the same practice as in [5], DNase-seq read counts are normalized per chromosome such that average read count per base are identical for all chromosomes. For each factor, we obtained the genomic coordinates of the top 100,000 motif matches genome-wide, and ranked these potential binding sites in terms of likelihood of differential binding. From this ranked list we computed prediction performance indicators including AUROC (area under receiver operating curve), AUPR (area under precision recall curve) and precision at given recall values.

The set of true differentially bound sites are constructed using matching factor and condition ChIP-seq data from ENCODE. This ground truth set is obtained by processing ChIP-seq experiments using multi-condition GEM [7] and edgeR [8] (more details in Methods). We combined information from both programs to set a stringent criterion for selecting ground truth sites. This ensures that high-confidence differential sites are retained so that performance metrics evaluated on the ground truth set are accurate.

We evaluate the performance of methods for calling differentially bound events by computing p-values for all AUPR and AUROC values. Because of the large size imbalance of the positive and negative sets in this task, we consider the precision value (equivalently the left side of the PR curve) to be a better performance indicator as well as more interesting biologically.

We compared several methods to rank motif containing candidate factor binding sites. The first method ranks the candidate sites according to the difference in normalized read counts over a 600bp window at each site between K562 and GM12878. We found that this method has an average precision of 9% at 10% recall and average AUPR of 0.056. This indicates that simply using imbalance in DNase-seq read counts is a poor predictor of differential binding.

Our second method ranks candidate binding sites according to the difference of PIQ [5] shape scores between K562 and GM12878. We reasoned that since PIQ shape scores capture the conformance of DNase read profiles to the characteristic factor hypersensitivity profiles, this would give rise to better classification performance. Indeed, the average AUPR for this method is 0.103, and the precision is 23% at 10% recall, which is a large improvement compared to the read count baseline.

**Fig 1:**
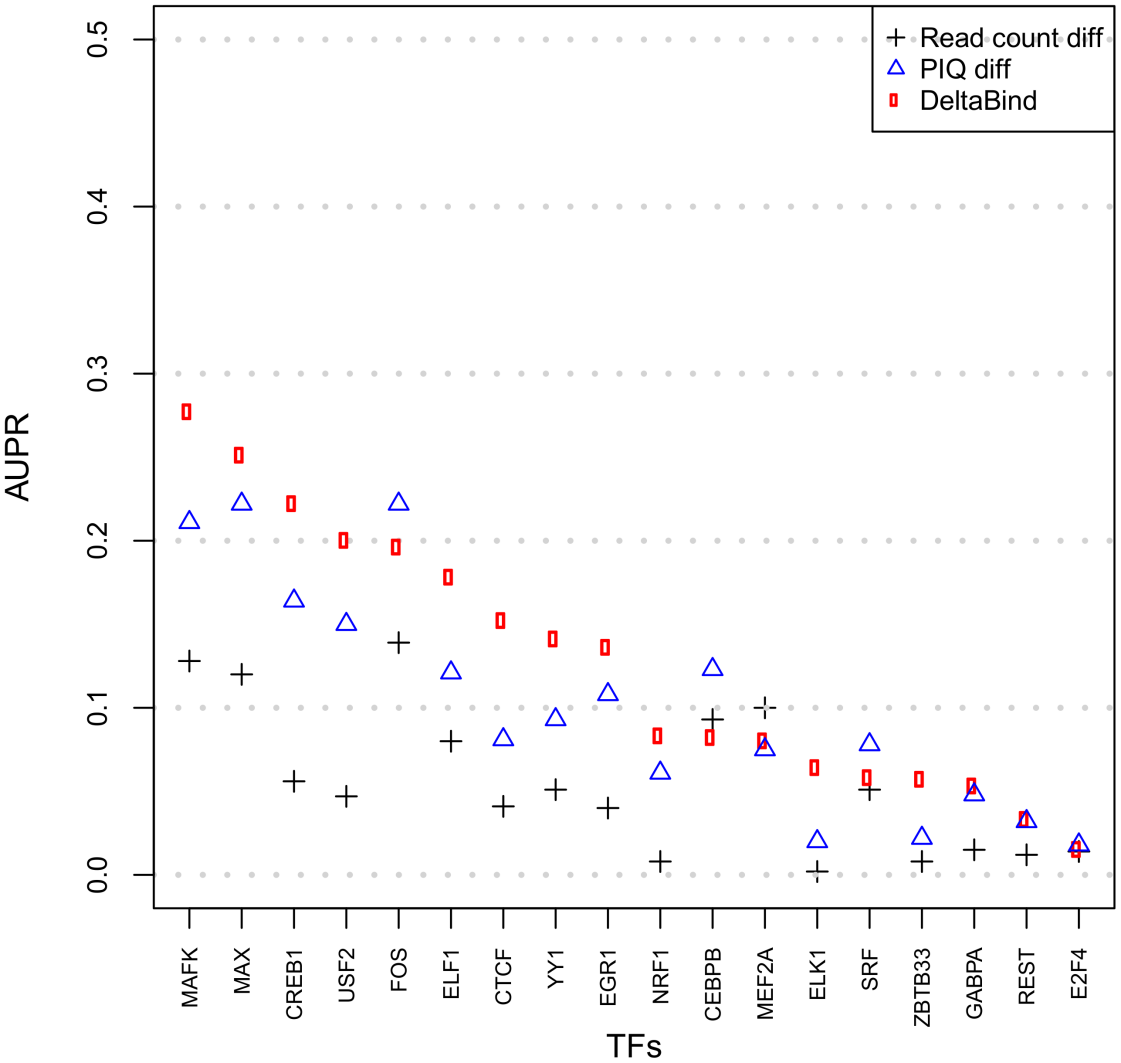
AUPR for 18 tested factors using 3 different methods.

Finally, we used DeltaBind to model differential binding from PIQ scores of two replicates experiments of each cell state (see Methods). This method achieves higher precision (28%) and AUPR (0.127). DeltaBind first transforms all PIQ shape scores to their respective ranks, and then estimates the probability of differential binding given the ranks of PIQ scores in both K562 and GM12878 experiments. Fig 1 shows the comparison of AUPR values for the three methods described above. PIQ score difference and DeltaBind have higher prediction power relative to read count difference. DeltaBind outperforms the other two in 13 out of 18 factors. The read count based method has the worst performance in 16 out of 18 factors.

We find that DeltaBind’s rank transformation typically greatly improves predictive power. In fact, the use of a rank transformation allows DeltaBind to be a general method for differential event detection, since it can be applied to any rank transformed scores of a given event across two conditions produced by any algorithm. For example, DeltaBind also improves prediction of differential occupancy using Centipede [4] outputs (Fig C and D in S1 File).

Our results also show that the accuracy of differential occupancy detection varies widely among factors. Using DeltaBind, we find that precision ranges from 5.3% to 65% at 10% recall, with an average of 28%. Therefore, while the average prediction precision is low, for some factors differential binding can be reasonably well predicted by DNase data, and for some other factors the prediction is very poor.

Table 1 summarizes information about the factors used in the prediction and the prediction performance. For each factor, the number of ground truth differential sites, number of candidate sites and their ratios are shown. For the prediction accuracy, AUPR, AUC and precision values are shown.

**Table 1:** Summary statistics of differential binding prediction for different factors.

factor name	Prop. of ChIP diff. sites	DeltaBind Precision	DeltaBind AUC All p<1e-4	DeltaBind AUPR All p<1e-4	PIQ diff AUPR	Reads diff AUPR	factor class
CEBPB	0.0017	0.184	0.844	0.082	0.123	0.093	
CREB1	0.0058	0.413	0.942	0.222	0.164	0.056	pioneer
CTCF	0.0132	0.187	0.903	0.152	0.081	0.041	pioneer
E2F4	0.0012	0.053	0.794	0.015	0.018	0.014	pioneer
EGR1	0.0077	0.242	0.887	0.136	0.108	0.04	pioneer
ELF1	0.0106	0.422	0.828	0.178	0.121	0.08	pioneer
ELK1	0.0001	0.285	0.978	0.064	0.02	0.002	
FOS	0.0038	0.358	0.951	0.196	0.222	0.139	migrant
GABPA	0.0011	0.18	0.817	0.053	0.048	0.015	pioneer
MAFK	0.0026	0.65	0.962	0.277	0.211	0.128	migrant
MAX	0.0032	0.534	0.916	0.251	0.222	0.12	settler
MEF2A	0.0029	0.263	0.707	0.08	0.075	0.1	migrant
NRF1	0.0015	0.138	0.957	0.083	0.061	0.008	pioneer
REST	0.0004	0.114	0.853	0.033	0.032	0.012	
SRF	0.0023	0.182	0.726	0.058	0.078	0.051	migrant
USF2	0.0025	0.402	0.965	0.2	0.15	0.047	settler
YY1	0.0079	0.359	0.865	0.141	0.093	0.051	migrant
ZBTB33	0.0023	0.08	0.928	0.057	0.022	0.008	pioneer

(P-values for DeltaBind AUC and AUPR for each factor are estimated by bootstrap; all are less than 1e-4.)

Differential binding of settler and migrant factors can be better predicted by DNase data than pioneer factors

**Fig 2:**
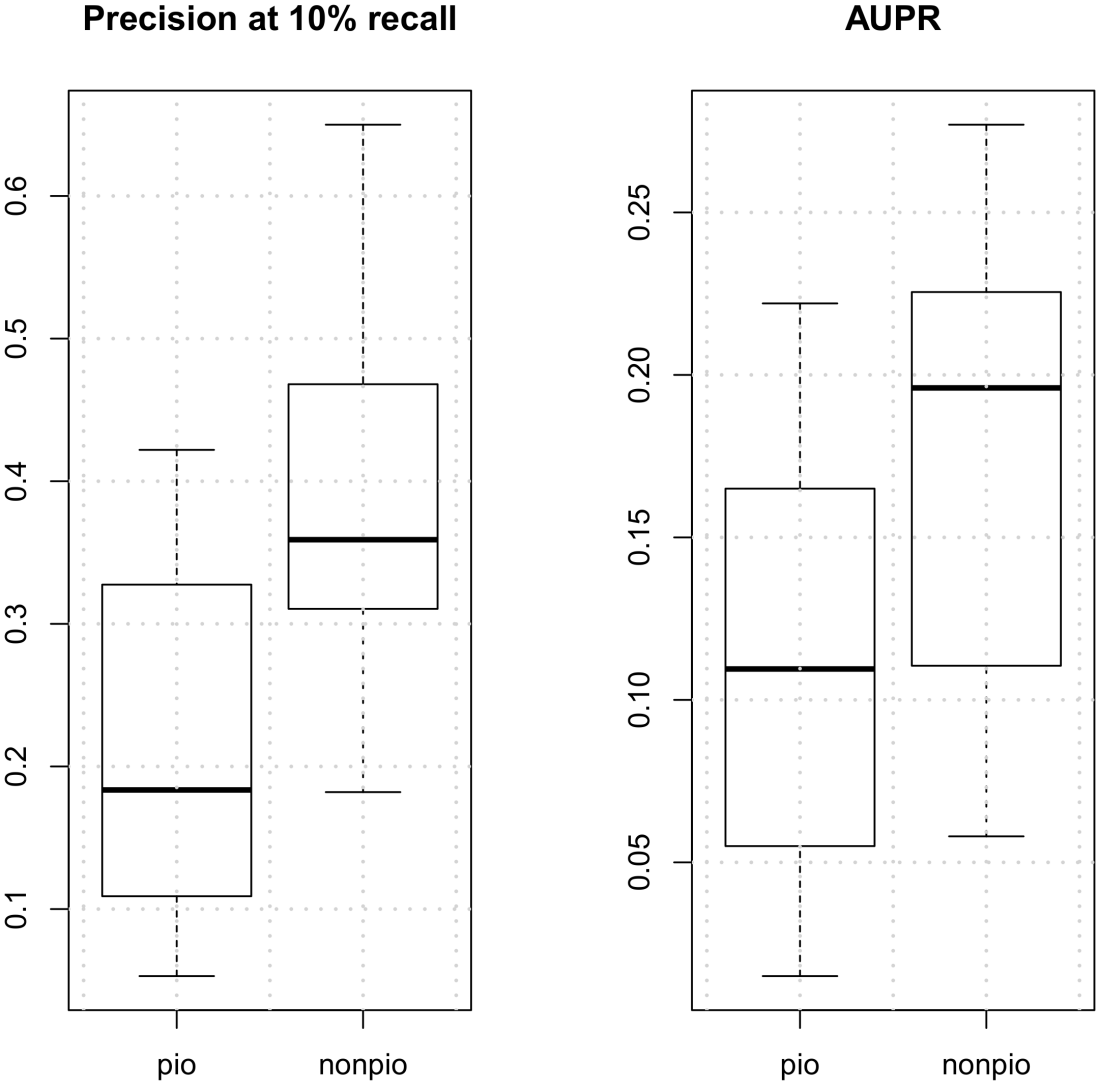
Comparison of prediction performance for pioneer class factors and non-pioneer (settler and migrant) class factors.

We hypothesized that since settler and migrant factors (non-pioneer factors) [5] bind open chromatin, the DNase profiles around binding events of these factors would have higher predictive power than those around pioneer factors which opens chromatin with a potentially more complex mechanism. In order to test this hypothesis, we identified 8 pioneer factors and 7 non-pioneer factors in our list of factors [5]. Fig 2 shows the boxplots of precision (10% recall) and AUPR for pioneer and non-pioneer factors. The mean precision is 39% for non-pioneer factors, significantly higher than pioneer factors with mean precision of 23% (p = 0.04). AUPR also provides the same insight, although in this case it is less discriminant than the precision metric. The mean AUPR is 0.17 for non-pioneer factors, compared to 0.12 for pioneer factors. Thus, differential binding prediction from DNase-seq data is more accurate for non-pioneer factors than pioneer factors.

## Materials and methods

### Data for CTCF binding in relation to allelic imbalance at SNP loci

Statistics of co-occurrence of CTCF binding and allelic imbalance are obtained from Supplementary Table 11 of [6].

### Data source and the generation of true ChIP-seq differential events

We ran and tested our methods on ENCODE Consortium DNase-seq experiments for K562 and GM12878 cells [9]. We chose these cells because they were also used to profile the binding a large and diverse set of factors. We evaluated the accuracy of our predictions with matching ChIP-seq experiments for both cell types to generate a list of high-confidence differential binding events that serve as our ground truth for prediction performance analysis. We designed a four-step pipeline to obtain a set of high-confidence differential ChIP-seq binding events.

In the first filtering step, we process all available ChIP-seq data (including all control antibody experiments) for all factors and replicates of GM12878 and K562 with GEM [7]. GEM discovers motifs for the factor in each cell type, and we discard factors whose motifs discovered in the two cell types do not agree, as well as factors whose motifs discovered in either cell type does not agree with JASPAR database. This filtering step retains 18 transcription factors that share consistent motifs for both ChIP-seq experiments and the JASPAR database (Table 1).

In the second step, we process the 18 matching ChIP-seq experiments with multi-condition GEM (a GEM adaptation of MultiGPS [10]). Multi-condition GEM assigns reads to each putative protein binding site, and makes a prediction of binding status for each. We record the assigned reads and binding status of each site. We then process the recorded read counts for all sites with edgeR to identify sites of differential binding. We check that the top differential sites inferred by multi-condition GEM agree with those inferred by edgeR. We go down the ordered list of differential sites from multi-condition GEM, and for each rank in the list, compute the proportion of matched sites with edgeR locally. We stop including sites into the ground truth set after the proportion becomes small (threshold is set at max proportion / 1.6).

In the third step, we use edgeR [8] to compute p-values for the read counts for all ChIP-seq peak sites. However, edgeR can only analyze the ChIP-seq experiment reads and is unable to account for differential reads that are present in ChIP-seq control experiments (GEM controls for these). We remedy this by combining the results from multi-condition GEM and edgeR to obtain our ground truth set. More specifically, we take the edgeR differential events with p-values < 0.05 and filter for the events which (1) have insignificant GEM q-value (-log10Q1<2.5) in GM12878, (2) have significant GEM q-value (-log10Q2>2.5) in K562 and (3) have reasonably large GEM q-value difference (-log10Q2 + log10Q1>0.5) between K562 and GM12878.

Finally, our final ground truth set is the filtered set of GEM differential sites that lie within 20 bp of a factor motif match site found by PIQ.

### DeltaBind

DeltaBind infers differential binding events from single-condition binding scores for each condition. DeltaBind requires two replicate experiments for the “bound” condition and at least one replicate for the “unbound” condition. DeltaBind is an unsupervised method that assumes data can be explained by a statistical model which can be decomposed into simpler conditional probability components, with one component representing the probability of a given site being bound in one condition and the second component representing the probability of the site being unbound in the other condition. It learns the parameters of these distributions from data, and then uses the learned model to estimate the probability of a particular site being differentially bound given the DNase scores in both conditions. The method standardizes the input binding scores to rank space and works primarily with ranks. (More detailed motivation and description of DeltaBind can be found in Supplementary materials.)

More specifically, suppose we have two DNase-seq experiment replicates each for K562 and GM12878, and we want to infer binding sites which are bound in K562 and unbound in GM12878. Let $R_{ir}^{j}$, 1 ≤ *i* ≤ *N*, *j* = "*G*" *or* "*K*", *r* = 1 *or* 2, be the rank of the PIQ shape score of binding site *i*, condition *j*, and replicate number *r*, where *N* is the number of candidate binding sites, *j* = "*G*" denotes a GM12878 value, *j* = "*K*" denotes a K562 value, and *r* indexes the replicates. Let $R_{i}^{j}=mean\left(R_{i1}^{j},R_{i2}^{j}\right)$ be the average rank of a binding site *i* in condition *j*, and *R*_*i*_ be the vector of all 4 ranks in two conditions and two replicates. DeltaBind estimates the probability

$$P(A_{i},B_{i}\;|\;R_{i}),$$

for each site *i*, where *A*_*i*_ is the event that site *i* is bound in K562, *B*_*i*_ is the event that site *i* is significantly more weakly bound in GM12878 than in K562. We interpret site *i* to be differentially bound when *A*_*i*_ and *B*_*i*_ both occur. We decompose the above probability into

$$P(A_{i},B_{i}\;\left|\;R_{i})=P(A_{i}\;\right|\;R_{i})\;*\;P(B_{i}\;|\;A_{i},R_{i}),$$

and model each part by assuming mixture models on the relevant subset of data. We note that *B*_*i*_ is not a subset of *A*_*i*._ There are sites that are unbound in both conditions but have large differences between their PIQ scores in K562 and GM12878, in which case *B*_*i*_ occurs but not *A*_*i*._ So *P*(*A*_*i*,_ *B*_*i*_ | *R*_*i*_) is actually not equivalent to *P*(*B*_*i*_ | *R*_*i*_).

The first part, *P*(*A*_*i*_ | *R*_*i*_), denotes the probability of a binding site *i* being bound in K562, and we estimate this by using the notion of reproducibility of DNase-seq ranks. Reproducibility is a concept introduced in [11] and characterizes an event which produces positively correlated scores in replicate experiments with high mean. Its counterpart, irreproducibility, characterizes events that produce uncorrelated scores in replicates with low mean. Let $A_{i}^{\prime }$ denote a reproducible event in K562. Using the framework in the IDR paper, we estimate $P\text{(}A_{i}^{\prime }\;|\;R_{i1}^{K},\;R_{i2}^{K})$ by transforming the rank values through a normal quantile function and fitting a reproducible and irreproducible cluster (Fig A in S1 File). We then use the reproducibility score to compute the binding probability *P*(*A*_*i*_ | *R*_*i*_) as a function of mean K562 ranks $R_{i}^{K}$.

In the second part, we estimate *P*(*B*_*i*_ | *A*_*i*_, *R*_*i*_), the probability of site *i* being weakly bound or unbound in GM12878 relative to K562. To estimate this value, we model the difference of PIQ ranks between GM12878 and K562 for reproducible binding sites for each K562 PIQ rank (a small window is used in implementation). For each K562 PIQ rank, we classify binding sites into one of three categories: no significant rank difference between two cell types, significantly lower ranks in GM12878 than in K562, and vice versa (Fig A in S1 File). An EM-like algorithm is used to determine the probability of belonging to each of the three categories, giving an estimate of the conditional probability *P*(*B*_*i*_ | *A*_*i*_, *R*_*i*_). Details for both parts above can be found in the Supplementary Information.

**Fig 3:**
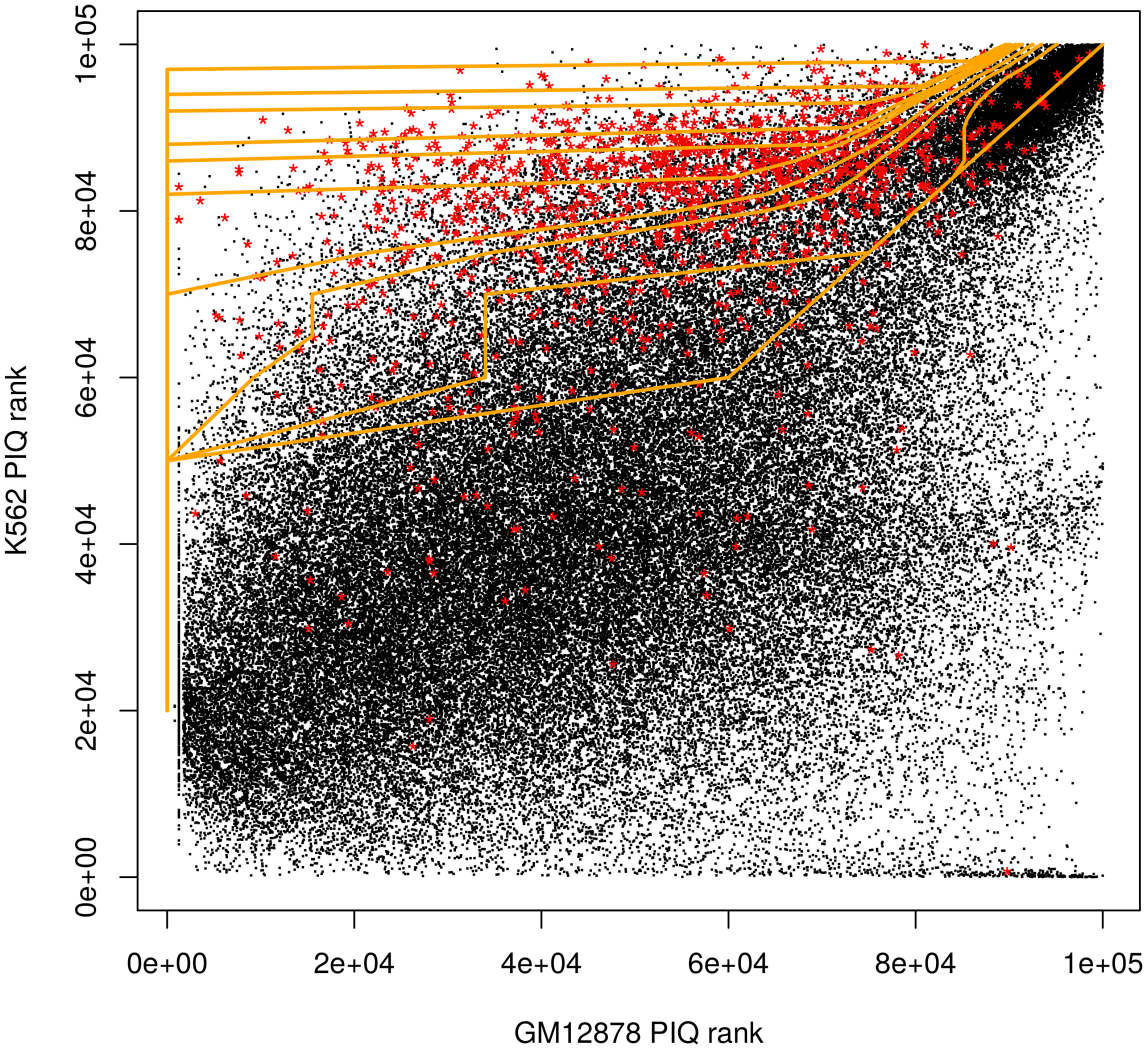
DeltaBind decision boundaries (orange) of different confidence levels. Axes are K562 PIQ rank vs. GM12878 PIQ rank. Red represents true differential sites indicated by ChIP-seq signals.

Finally, taking the product of *P*(*A*_*i*_ | *R*_*i*_) and *P*(*B*_*i*_ | *A*_*i*_, *R*_*i*_) gives an estimate of the probability of differential binding for site *i*. A set of decision boundaries for CTCF derived by this probability score is shown in Fig 3 (orange). Supplementary Fig 2 shows DeltaBind PR and ROC curves for several factors.

## Discussion

We have found that chromatin accessibility data cannot predict differential transcription factor occupancy with high precision, and the observed precision is factor dependent. Our results on K562 and GM12878 cells show that, on average, DNase-seq experiments provide 28% precision for known differential binding. Settler and migrant factors are generally more predictable, with precisions up to 65%, whereas pioneer factors are overall less predictable. We also showed that DNase imbalance at SNPs is not a good predictor of transcription factor binding state across cell types or conditions.

We note that the factors we considered do not exhibit extensive differential binding in K562 and GM12878. Thus predicting differential binding is a more difficult task than predicting binding in each cell type. For CTCF, only 1.32% of sites were classified as being bound in K562 and unbound in GM1287. As a consequence, DeltaBind predicts these events with 20% precision, while PIQ predicts binding in each cell type with 80% precision.

Finally, to analyze differential binding we developed a new unsupervised classifier DeltaBind that improves differential binding prediction accuracy from DNase-seq data with respect to the null model (30x better AUPR) and a read-count based method (2.3x better AUPR). DeltaBind can used to predict condition specific binding from any single condition binding predictor that outputs a score. We found that DeltaBind improves prediction accuracy for both PIQ and Centipede against other baseline approaches.

## Supporting information

S1 File Supplementary materials. (PDF)

S1 Dataset DNase-seq and ChIP-seq data tables for DeltaBind. (ZIP)
