# Supplementary material for: Differential chromatin profiles partially determine transcription factor binding
Source: PLoS One. 2017 Jul 13;12(7):e0179411. doi: 10.1371/journal.pone.0179411 (PMC5509100; doi:10.1371/journal.pone.0179411)
Supplement: S1 File — (PDF) [file pone.0179411.s001.pdf]

Supporting information

DeltaBind

DeltaBind infers differential binding events from single-condition binding scores for each condition. DeltaBind requires two replicate experiments for the "bound" condition and at least one replicate for the "unbound" condition. DeltaBind is an unsupervised method that assumes data can be explained by a structure of conditional probabilities based on data, with one component representing the probability of a given site being bound in one condition and the second component representing the probability of significantly differential events in the other condition. It learns the parameters of these distributions from data, and then uses the learned model to estimate the probability of a particular site being differentially bound given the DNase scores in both conditions. The method standardizes the input binding scores to rank space and works primarily with ranks.

We find that it is infeasible for an unsupervised method to model the conditional probability of differential binding directly, since we know neither the prediction accuracy of the single-condition scores, nor the size of the true ground truth differential ChIP-seq events. However, it is possible to model the joint probability (given DNase scores) of an event being reproducible in one condition and significantly more weakly bound in the other condition. We find that this can be used as a good surrogate for probability of differential binding, and provides a good score metric to rank candidate binding sites in terms of their likelihood of differential binding.

Suppose we have two DNase-seq experiment replicates each for K562 and GM12878, and we want to infer binding sites which are bound in K562 and unbound in GM12878. Let R_{ir}^{j} , $1 \leq i \leq N, j = "G"\ or\ "K", r = 1\ or\ 2$, be the rank of the PIQ shape score of binding site i, condition j, and replicate number r, where N is the number of candidate binding sites, $j = "G"$ denotes a GM12878 value, $j = "K"$ denotes a K562 value, and r indexes the replicates. Let $R_i^j\ =\ mean(R_{i1}^j, R_{i2}^j)$ be the average rank of a binding site i in condition j, and R_i be the vector of all 4 ranks in two conditions and two replicates. Let A_i be the event that site i is bound in K562, and B_i be the event that site i has a significantly weaker DNase profile in GM12878 than K562. Thus, we aim to model the conditional probability,

$$P(A_i, B_i \mid R_i),$$

for each site i. We decompose this into

$$P(A_i, B_i \mid R_i)\ =\ P(A_i \mid R_i)\ *\ P(B_i \mid A_i, R_i),$$

and model each component part by assuming mixture models on the relevant subset of data.

The first part, $P(A_i \mid R_i)$, denotes the probability of a binding site being bound in K562. First we note that it does not depend on GM12878 ranks, so $P(A_i \mid R_i) = P(A_i \mid\ R_{i1}^K, R_{i2}^K)$. As mentioned above, we model this probability by resorting to a measure of reproducibility. Reproducibility is a concept introduced in (Li, Brown, Huang, & Bickel, 2011)] and characterizes an event which produces positively correlated scores in replicate experiments with high mean. Its counterpart, irreproducibility, characterizes an event which produce uncorrelated scores in replicates with low mean. Let A_i' denote a reproducible event in K562. Then A_i' does not depend on GM12878 scores, so $P(A_i' \mid R_i)\ =\ P(A_i' \mid R_{i1}^K, R_{i2}^K)$. Using the framework in the IDR paper, we estimate $P(A_i' \mid R_{i1}^K, R_{i2}^K)$ by transforming the rank values through a normal quantile function and fitting a reproducible and irreproducible cluster (**Fig. S1a**). To improve robustness against noise in DNase scores and hence ranks, we compute the binding probability as a function of mean K562 ranks R_i^K. This is done by averaging across all binding sites with identical mean rank R_i^K under the IDR model, i.e., $P(A_i \mid R_i^K = r) = \int P(A_i' \mid R_{i1}^K, R_{i2}^K)\ \mathrm{d}P(R_{i1}^K, R_{i2}^K \mid R_i^K = r)$. This average can be estimated numerically using the fitted joint distributions of the IDR model for each small window of $R_i^K = r$. In practice, we

find that it is important to have fine window sizes for high values of r as they correspond to bound K562 sites; for low values of r, we use coarser windows.

In this process, we made the assumption that reproducibility of K562 DNase rank is a good surrogate for the actual binding of the transcription factor. This is reasonable because we expect strongly bound sites to have higher reproducible DHS shapes, and weak or unbound sites to generate random DHS shapes.

In the second part, we estimate $P(B_i \mid A_i, R_i)$, the probability of site i belonging to a weaker-than-typical class of binding sites in GM12878. We reason that this probability depends on the event A_i, the rank of site i in K562, R_i^K, and the difference of ranks between GM12878 and K562, $R_i^G - R_i^K$. We estimate $P(B_i \mid A_i, R_i)$ by first splitting R_i^K into a fine sequence of values covering its range, and then estimating a model of $P(B_i \mid A_i, R_i)$ for each fixed R_i^K value. For each R_i^K (within a small window), we fit a mixture model for the observed values of $R_i^G - R_i^K$.

Conditioning on A_i can be approximated well for strong K562 binding sites by selecting binding sites close to the diagonal on the R_{i1}^K vs R_{i2}^K plot, since sites on this diagonal has high probability of belonging to the reproducible component in the IDR model. This approximation is very good for highly ranked K562 binding sites, since the reproducible component dominates there, and thus provides high fidelity for the events we are most interested in. **Figure S1b** shows a typical plot of $R_i^G - R_i^K$ for a high K562 rank (black curve). The plot shows a concentrated cluster of "typical" values for the rank difference around 0, and a heavier left tail of sites with much lower ranks ("atypical values") in GM12878. We use a mixture model to capture this overall distribution, consisting of three components, a "typical" component in the center around 0, an "atypical left" component and an "atypical right" component (insignificant for this window of R_i^K). For simplicity, we model the two tail components as uniform distributions and the center component Gaussian. Results show that this simplification works reasonably well. Then, we estimate model parameters with an EM-like algorithm to iteratively assign sites to each cluster and refine parameters.

Finally, taking the product of the two probabilities gives an estimate of the probability of differential binding for site i. Then, decision boundaries at any specified differential binding probability can be defined. A set of decision boundaries derived by this probability score is shown in **Figure S1c** (orange). We have enforced that decision boundaries are increasing and lie above the diagonal. Axes are average K562 rank vs. average GM12878 rank. The red points are the ground truth sites.

The DeltaBind software is available at: bitbucket.org/deltabind_cgs/deltabind

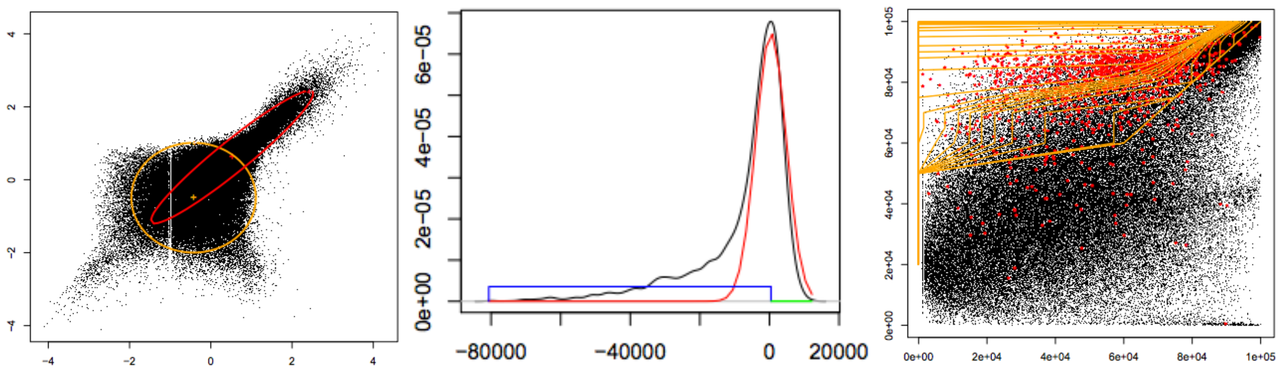

(a) (b) (c)

Figure S1: DeltaBind model fitting. (a) Fitting reproducible (red) and irreproducible (orange) clusters of binding sites based on PIQ shape score. (b) Fitting the conditional model of differential binding, black: data distribution, red: non-differential component, blue: differential component with lower ranks in GM12878 than K562, green: differential component with higher ranks in GM12878 than K562. (c) Decision boundaries (orange) of different confidence levels. Axes are K562 PIQ rank vs. GM12878 PIQ rank. Red represents true differential sites indicated by ChIP-seq signals.

PR and ROC curves for selected TFs.

Figure S2 shows the PR and ROC curves of some of the well-performing factors that we have identified in our dataset. The dashed line represents the linear diffPIQ classifier which prioritizes candidate sites by the difference between K562 shape score and GM12878 shape score. We note that while AUPR is relatively low for all TFs, AUROC is generally high for most TFs (values in Table 1).

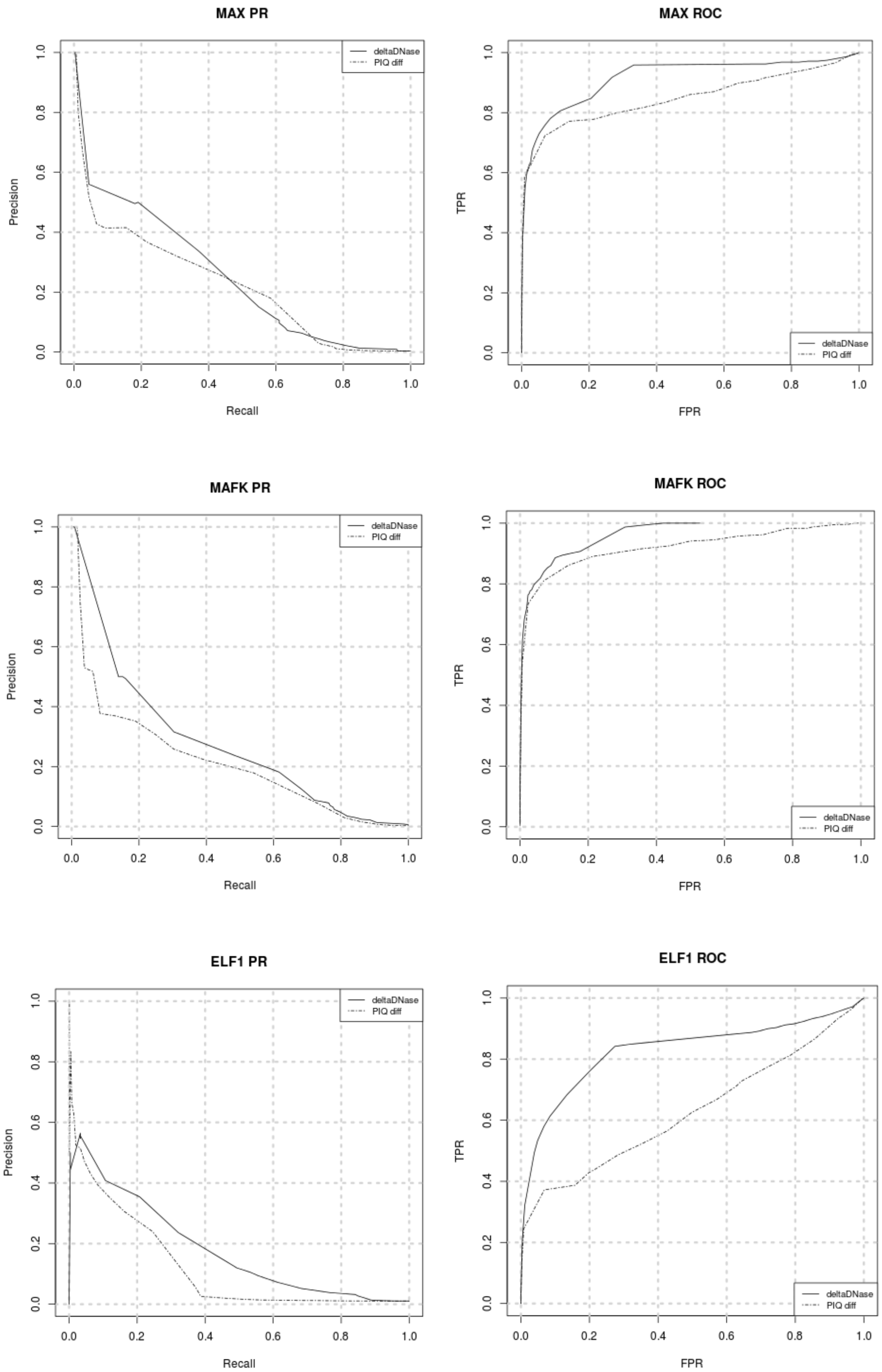

Figure S2: PR and ROC curves for selected TFs.

DeltaBind improves differential binding prediction from Centipede scores

Centipede uses a hierarchical Bayesian mixture model to infer the posterior probability of transcription factor binding from single condition DNase-seq experiments. We first explored whether we can use Centipede outputs for K562 and GM12878 to detect differential factor occupancy. Let Pki be the posterior probability of a site i in K562, and Pgi be that in GM12878, we tested three methods to combine scores from the two cell types into a score for ranking differential binding (bound in K562 and unbound in GM12878):

$Pki * (1 - Pgi)$ product of probability of being bound in K562 and probability of not bound in GM12878

$Pki - Pgi$ difference of probability

$log\ Pki - log\ Pgi$ difference of log probability

We find that DeltaBind applied to PIQ output achieves higher prediction accuracy than all three Centipede scoring methods for all eighteen factors tested. **Figure S3** shows the AUPR for DeltaBind and the best Centipede method for each factor.

We next tested applying DeltaBind to Centipede's posterior binding probabilities jointly, by converting them into rank space. Our results show that DeltaBind is able to improve the prediction accuracy compared to all three Centipede scoring methods above, demonstrating the generality of the DeltaBind method. Overall, DeltaBind has the best prediction performance when applied to PIQ outputs. (**Fig. S4**)

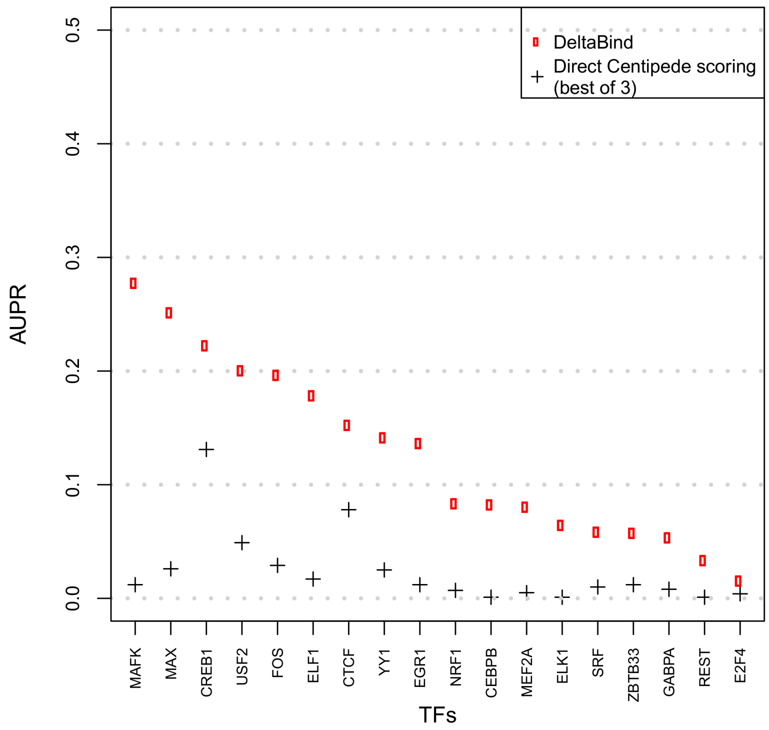

Figure S3. Comparison of DeltaBind prediction performance with best Centipede scoring method.

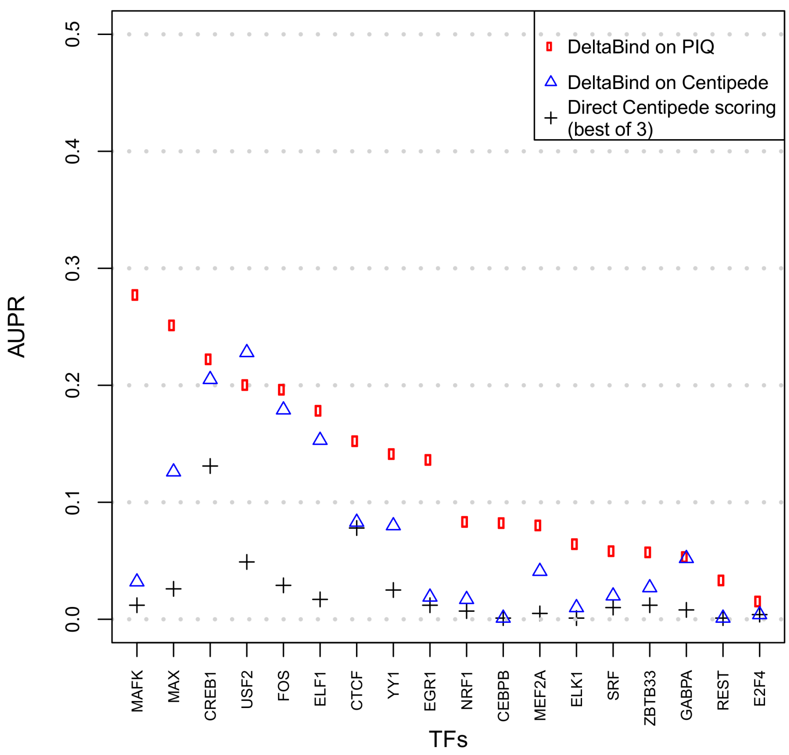

Figure S4. DeltaBind applied to Centipede outputs improves differential binding prediction.

Statistics of differential binding inference for transcription factors

Table S1 shows the relevant statistics of the differential binding inference task for all transcription factors studied. Specifically, the table columns show, in order, factor name, number of candidate binding sites, number of true differentially bound sites evidenced by ChIP-seq data (true positives), the proportion of the true positives, DeltaBind precision, AUC and AUPR.

Table S1. Statistics of differential binding inference for different factors.

factor name	Total binding sites	No. of true pos	Prop. of ChIP diff. sites	DeltaBind Precision	DeltaBind AUC	DeltaBind AUPR
CEBPB	97731	169	0.0017	0.184	0.844	0.082
CREB1	30251	176	0.0058	0.413	0.942	0.222
CTCF	100000	1321	0.0132	0.187	0.903	0.152
E2F4	100885	126	0.0012	0.053	0.794	0.015
EGR1	99999	772	0.0077	0.242	0.887	0.136
ELF1	99339	1056	0.0106	0.422	0.828	0.178
ELK1	97805	13	0.0001	0.285	0.978	0.064
FOS	99452	373	0.0038	0.358	0.951	0.196
GABPA	68877	77	0.0011	0.18	0.817	0.053
MAFK	90713	237	0.0026	0.65	0.962	0.277
MAX	98143	315	0.0032	0.534	0.916	0.251
MEF2A	99998	290	0.0029	0.263	0.707	0.08
NRF1	99785	146	0.0015	0.138	0.957	0.083
REST	100000	44	0.0004	0.114	0.853	0.033
SRF	100000	233	0.0023	0.182	0.726	0.058
USF2	97624	247	0.0025	0.402	0.965	0.2
YY1	99931	793	0.0079	0.359	0.865	0.141
ZBTB33	100000	231	0.0023	0.08	0.928	0.057
